# Pathways between intimate partner violence and HIV care and treatment during pregnancy and postpartum: A qualitative study in southwestern Kenya

**DOI:** 10.1371/journal.pgph.0004163

**Published:** 2025-09-03

**Authors:** Leah Schrubbe, Catherine Makokha, Liza Kimbo, Anna Helova, Zachary Kwena, Lynae Darbes, Moses Okombo, Evelyne Owengah, Clara Calvert, Heidi Stöckl, Elizabeth A. Bukusi, Janet M. Turan, Abigail M. Hatcher

**Affiliations:** 1 Department of Population Health, Faculty of Epidemiology and Population Health, London School of Hygiene & Tropical Medicine, London, United Kingdom; 2 Centre for Microbiology Research, Kenya Medical Research Institute, Nairobi, Kenya; 3 Department of Health Policy and Organization, School of Public Health, University of Alabama at Birmingham, Birmingham, Alabama, United States of America; 4 Department of Health Behavior and Biological Sciences, School of Nursing, University of Michigan, Ann Arbor, Michigan, United States of America; 5 Usher Institute, University of Edinburgh, Edinburgh, United Kingdom; 6 Institute for Medical Information Processing, Biometry and Epidemiology, Faculty of Medicine, LMU Munich, Munich, Germany; 7 Department of Health Behavior, Gillings School of Global Public Health, University of North Carolina at Chapel Hill, Chapel Hill, North Carolina, United States of America; 8 School of Public Health, Faculty of Health Sciences, University of the Witwatersrand, Johannesburg, South Africa; Ben-Gurion University of the Negev, ISRAEL

## Abstract

Intimate partner violence (IPV) is associated with suboptimal HIV treatment behaviors and health outcomes among perinatal women. Less is known about the postpartum phase or how distinct types of perinatal IPV exposure may inhibit HIV-related care. We conducted a qualitative study nested within an ongoing trial among perinatal women in rural Kenya to explore the influence of IPV on adherence to HIV treatment during pregnancy and postpartum. In 2022, a trained researcher fluent in Dholuo and Kiswahili conducted 23 semi-structured interviews with women up to 2 years postpartum living with HIV and self-reported IPV in their current relationship. Digitally recorded interviews were translated into English, transcribed verbatim, and thematically coded using deductive and inductive techniques. Nearly all women reported psychological and financial IPV, the majority reported physical IPV, half reported male controlling behaviors, half reported reproductive coercion, and many reported sexual IPV. Many women described a link between IPV and their adherence to perinatal HIV care and treatment. An indirect pathway was exhibited when psychological IPV heightened mental distress, leading to non-adherence through symptoms of depression and anxiety. A second path occurred when financial IPV and withholding food led to HIV treatment challenges. A direct pathway occurred when male partners sabotaged HIV treatment or controlled women’s access to HIV care. In turn, women’s evasion of IPV through leaving home or strategic non-disclosure had unanticipated consequences for their HIV treatment. Despite enduring IPV, many women described adhering to HIV treatment to sustain good health for themselves and their children. IPV-exposed women living with HIV described multiple ways a violent relationship was detrimental to maintaining their HIV treatment. To meet global goals to end vertical transmission of HIV and improve maternal and infant health, preventing and addressing IPV within maternal health settings should be prioritized in HIV programming.

## Introduction

Globally, perinatal women living with HIV (WLH) continue to face challenges with antiretroviral therapy (ART) adherence despite expanded treatment access. A cross-sectional analysis of population-based data from nine sub-Saharan African countries (2015–2018) showed that the prevalence of suboptimal ART adherence among pregnant and breastfeeding women was 20.1% (95% CI: 15.7-24.5%) [1]. Suboptimal adherence may have risen further as health systems altered priorities and resources during the COVID-19 era [2, 3]. Kenya, one of 17 countries prioritized in ‘The Global Alliance to End AIDS in Children by 2030’ [4], is a country with approximately 1.4 million people living with HIV [5]. An estimated 90% of pregnant WLH in Kenya received ART for prevention of vertical transmission in 2022 [5]. Although vertical transmission rates have decreased over time, an estimated 8.6% of infants vertically acquired HIV in 2022 [5].

Intimate partner violence (IPV) is a social determinant of poor ART adherence and unsuppressed viral load in women, including perinatal women [1, 6–11]. Longitudinal prospective cohort data among perinatal women from South Africa showed physical, sexual, and/or psychological IPV was associated with halved odds of postpartum viral suppression [11], as well as halved odds of optimal ART adherence at any time during the perinatal phase [10]. Evidence from across sub-Saharan Africa suggests IPV during pregnancy is common, with an overall estimated prevalence of 15.2% (95% CI: 14.4-16.1%) in a meta-analysis [12]. Kenyan population-based data from 2014 shows 9.2% of women experience physical, sexual, and/or psychological IPV during pregnancy [13].

IPV occurs in forms that are physical, sexual, psychological, or financial (includes economic control, economic exploitation, employment sabotage, and refusal to contribute/ male economic irresponsibility) [14]. Extant IPV literature often focuses on measuring and reporting physical and/or sexual violence [15] and these types of IPV are associated with worse maternal and neonatal health [16, 17]. There is less research on other types of perinatal IPV such as psychological and financial, but these also alter mental and physical health outcomes [18–23]. Reproductive coercion is generally understood to refer to pregnancy coercion, contraceptive sabotage, and controlling the outcome of a pregnancy [24]. Reproductive coercion and partner control similarly harm maternal health [25, 24], yet these forms of IPV have been insufficiently examined in HIV literature. Pregnancy can be a time of increased vulnerability for IPV due to the physical, social, and economic demands of this phase [26, 27]. A Kenyan qualitative study found pregnant women felt particularly vulnerable to IPV due to their economic dependency on male partners [28]. In other qualitative evidence, pregnant South African WLH reported certain aspects of IPV became more frequent and severe during their pregnancy, particularly controlling behavior [29].

Qualitative and quantitative evidence have explored several pathways linking IPV to HIV outcomes among pregnant and postpartum women [10, 30–33]. These include mental health [10, 30–32], partner non-disclosure of HIV status [30, 31], social isolation [31], lack of partner support for ART [30], withholding food and money [30, 33], and partner control [31]. However, there is a lack of understanding on how different types of IPV may impact women’s HIV care and treatment in unique ways. We conducted a qualitative study nested within an ongoing three-arm couple-randomized controlled trial called Jamii Bora (which means “better families” is Kiswahili), among perinatal women in rural Kenya to explore how and why certain types of IPV may influence HIV treatment during pregnancy and in the postpartum period.

## Methods

### Study setting

Women in the parent Jamii Bora trial were recruited from 24 antenatal care (ANC) clinics in Kisumu and Migori Counties of southwestern Kenya. These counties are among the top four highest HIV-burden counties [34] and account for 13% of new infant HIV infections in Kenya [35]. Clinics provide integrated ANC and HIV care, and pregnant and breastfeeding women who newly test positive for HIV are offered lifelong, immediate treatment. This region has the highest prevalence of IPV in Kenya with 57% of women aged 15–49 having ever experienced physical IPV and 32% having ever experienced sexual IPV [36]. Evidence shows IPV during pregnancy in this region is common. A cross-sectional study conducted at Kisumu County Hospital found the prevalence of physical, sexual, and/or psychological IPV was 37% during pregnancy [37].

### Trial study design

We conducted a nested qualitative sub-study in the Jamii Bora trial (clinical trials registration: NCT03547739). The trial was conducted 2019–2024 among 800 pregnant women (by design, 2/3 HIV-positive and 1/3 HIV-negative at baseline) and their male partners as well as 167 non-randomized women. The trial aimed to examine testing strategies for couple engagement in vertical transmission of HIV and family health [38]. Couples were randomized to one of three groups: 1) home-based couple counselling (intervention arm), 2) couples’ HIV self-testing, or 3) standard of care (male partner clinic invitation letters) and followed until 18 months postpartum. Women in the parent trial who reported severe IPV in the past six months at baseline were not randomized and their male partners were not contacted to ensure safety. They were, however, invited to participate individually in study assessments up to 18 months postpartum. All trial participants provided written informed consent and were compensated for completing assessments.

### Sampling and recruitment

Women from any arm, including non-randomized women, of the Jamii Bora trial were invited to participate in this qualitative sub-study if they met eligibility criteria. Women were eligible to participate in a semi-structured interview if they were living with HIV, reported IPV (physical, sexual, and/or psychological) during the current relationship (either before, during, or after pregnancy) on the study questionnaire at any timepoint, and were ≤24 months postpartum [39, 40]. A total of 29 participants met these inclusion criteria and were contacted by the study team, and 23 were reached and agreed to take part. Of the 23 women, a total of eleven were not randomized in the Jamii Bora study, three were in the home-based couple counselling group, four were in the couples’ HIV self-testing group, and five were in the standard of care group. They were all enrolled in the parent Jamii Bora study in 2020 or 2021.

### Data collection and management

The interview guide included such topics as pregnancy, HIV partner disclosure, intimate partner violence, prevention of vertical transmission of HIV, and HIV care and treatment. Study documents were translated and back-translated by a local researcher into Kiswahili and Dholuo. We hired an experienced qualitative interviewer from the local area, who spoke all three relevant languages, was familiar with the local context, and had experience interviewing women about their experiences with IPV. The interviewer participated in a training which included education on violence against women and how to interview women on IPV. We conducted weekly check-in calls with the interviewer to discuss recruitment, debrief on completed interviews, discuss emerging themes, and address any challenges and/or questions. The interviewer wrote up field reports for each interview. Data collection was conducted from 3 June 2022 – 3 August 2022.

All sub-study participants met with a qualitative interviewer in a private space at a local health facility. Informed written consent and semi-structured interviews were conducted in the participant’s preferred language (Kiswahili, Dholuo, or English) and audio recorded with participant permission. Participants were compensated with 500 Kenyan shillings (approximately $3 USD) for their transportation expenses. Interviews were transcribed and, if necessary, translated into English by one of two multilingual, qualitative interviewers who participated in the training.

Data were stored securely to ensure participant’s privacy and confidentiality. All participants provided written, informed consent and the original signed, paper consent forms were stored in a locked file cabinet with the audio recorder and handwritten fieldnotes. Once interview audio files were uploaded to a secure password-protected network drive and receipt verified immediately after the interview, they were deleted from the audio recorder. Only approved research staff had access to study files and data. After transcription, participants were given pseudonyms and all identifying information was removed. Any files with identifying information were encrypted.

### Data analysis

Preliminary analysis began with the initial review of transcripts and a team approach to analysis comprised coding discussion meetings and double-coding. All interview data were coded in Dedoose version 9.2.006 (2024, SocioCultural Research Consultants, LLC, Los Angeles, CA) by the first author and a portion of the transcripts were double coded by the second author. Data were analyzed using a thematic analytical approach in which data was deductively and inductively coded based on prior literature, identified themes within the interview guide, and emerging themes from interviews [41]. An initial coding framework was developed based on the interview guide and initial ideas around the data. Utilizing this coding framework of ‘broad codes’, data were coded using thematic coding to identify chunks of text related to each code [42]. Thematic codes comprised topics, or ‘thematic baskets’, such as “types of IPV experienced,” “change in IPV over time,” or “HIV status disclosure”. Observing the patterns and themes arising from the data during initial coding and discussion led to a second, inductive fine coding. Drawing from participant’s views and experiences, fine codes were developed to build meaning around each theme. Researchers then met to discuss results and build consensus around the fine coding.

### Ethical considerations

Original ethical approval for the Jamii Bora study in Kenya was obtained from the institutional review board (IRB) of the Kenya Medical Research Institute (KEMRI) (KEMRI SERU 3710), the University of Alabama at Birmingham (UAB) (IRB-300001427), and the University of North Carolina at Chapel Hill (IRB# 20–2198). Ethical approval for this qualitative sub study of Jamii Bora was obtained from the Research Ethics Committee at the London School of Hygiene and Tropical Medicine (Ethics Ref: 26557), the KEMRI (amendment to KEMRI SERU 3710), and UAB (amendment to IRB-300001427). Fieldworkers were trained in human subjects research ethics. Additional information regarding the ethical, cultural, and scientific considerations specific to inclusivity in global research is included in the Supporting Information (S1 File).

Ethical considerations for researching violence against women were followed [43]. Strict procedures were implemented to ensure participant’s informed consent, confidentiality, and safety. To minimize perceived distress, women were free to take breaks during the interview if needed, or to stop the interview at any time. The interviewer was also trained to change the topic of conversation during the interview should someone interrupt the privacy needed to conduct the interview. The interviewer was trained to follow the standard operating procedures for addressing cases of severe IPV used during the trial, which included providing a tailored local resource sheet hidden inside a tissue packet and specific contacts at nearby health facilities. The resource sheet included contact information for healthcare providers, including mental health and pastoral services, as well as local community organizations providing assistance with counseling, legal, and family support services. All participants in the parent study who experienced IPV were offered tailored, warm referrals by a counselor and these interactions were followed using an adverse reporting protocol until the IPV resolved. The qualitative interviewer was also trained to assist in scheduling onwards referral appointments if desired by the participant, but none of the participants opted for this. Our protocol called for documenting all cases of current, severe IPV or current suicidality using social harms and adverse reporting procedures of the parent trial, which included reporting to local and international ethical boards as well as an external Data Safety and Monitoring Board. As interviews progressed, we did not detect any new cases of severe IPV. We did respond to one case of suicidality, which was documented using the adverse reporting plans of the parent study and followed up by a study counselor trained in managing participant distress and offering referrals for mental health and IPV.

## Results

### Sample characteristics

Of the 23 women interviewed, the median age at baseline (when enrolled into Jamii Bora) was 27, the majority of women had only a primary school education, and over one third had been diagnosed with HIV in the past 1–2 years (Table 1). Most had been on ART prior to the perinatal phase, but 30.4% started treatment during this pregnancy. The mean number of months postpartum at the time of the qualitative interview was 13 (range: 4–24).

**Table 1:** Sample characteristics at baseline in Jamii Bora study^*^ (n = 23).

Characteristic	Number (%)	Median (Range)
**Sociodemographic**		
Age (years)		27 (19-40)
Parity		3 (0-8)
Highest level of education: some primary or finished primary school	16 (69.6%)	
No household electricity	12 (52.2%)	
Has own mobile phone	16 (69.6%)	
Household food insecurity^#^	11 (47.8%)	
**Relationship-related**		
Married	23 (100%)	
Partner’s age (years)		36 (23-50)
Length of relationship (years)		4 (0-20)
Husband has additional wives	4 (17.4%)	
**HIV-related**		
First tested HIV+ recently (2020–2021)^^^	9 (39.1%)	
Started ART during most recent pregnancy	7 (30.4%)	

*The baseline timepoint was < 36 weeks’ gestation

#Defined as no food to eat of any kind in the house because of lack of resources, in the past four weeks

^Participants were initially enrolled into the Jamii Bora study between 2020 and 2021

During the interview, the majority of women described experiencing physical, psychological, and financial IPV in their male partner relationships. Approximately half reported reproductive coercion, half reported male controlling behaviors, and many reported sexual IPV. Most women described experiencing multiple types of IPV over the course of their current relationship, the majority of which occurred perinatally, but also before and after pregnancy.

### Pathways between IPV and HIV care and treatment

Many women described a link between IPV and their challenges with perinatal HIV care and treatment. The first theme was IPV exposure worsens HIV care and treatment indirectly through effects on mental health. A second theme was financial IPV indirectly leads to challenges with taking ART through denied access to money and food. Thirdly, IPV included behaviors that directly sabotaged HIV treatment or access to clinical care. The fourth theme was safety behaviors to avoid IPV have unanticipated consequences for HIV care and treatment. Lastly, we found a protective pathway of resiliency and commitment to HIV treatment for women and their children.

Below, we detail these pathways alongside illustrative quotes and descriptions of women’s stories (names have been pseudonymized). Though these specific pathways were identified, women’s experiences sometimes overlapped between pathways.

### IPV exposure worsens HIV care and treatment via adverse impacts on mental health

Though most women described experiencing depressive symptoms either past or present, some women specifically described how the impact of IPV on their mental health impacted their ART adherence. One 23-year-old woman, Kioni, described experiencing physical, sexual, psychological, and financial IPV as well as reproductive coercion from her partner. After disclosing her HIV-positive status to her partner, he physically abused her, accused her of infidelity, and blamed her for acquiring HIV. Afterwards, he continued to blame, belittle, and insult her. She explained that the insults related to her HIV-status affected her mental health, adding to the depression she was already feeling as a result of her diagnosis. She had fallen out of HIV care several months prior to the interview and was off ART. She described how her partner’s constant insults contributed to her poor ART adherence:

When he is drunk, he insults me that I am taking drugs (*ART*). I then tell him what will happen if the people in the village know about it because he keeps telling me about the fact that I am on drugs all the time…I told him that I will take my children to our place if I become too sick. I can even tell my brother to take them (*the children*) and stay with them and leave him to stay in peace. He has since ceased reminding me that I am on drugs. In fact, he has contributed to me not adhering to the drugs because he ridicules me when I am taking drugs.

-Kioni, 23 years, not randomized in trial

Amelia, a 21-year-old woman, described how her partner’s psychological abuse and financial abuse were intertwined. She explained how this “invisible” IPV resulted in poor physical and mental health and how she hid it from others:

I had lost so much weight. Most people who knew me could tell that something was wrong even though I would deny it…Since my husband never beat me even once, they were wondering what the matter was. It is possible not to be physically assaulted but then the emotional abuse is enormous. This is manifested in the way that he replies to me when I ask him anything…They would insist my case was too bad and I needed to reduce my stress. Of course, I was fine while conversing with people but as soon as they leave, my worries resume…I would remember the things that he was telling me, and tears would just drop down my cheeks. I almost lost my mind.

-Amelia, 21 years, randomized in trial

Insulted for infertility issues, barred from getting a job due to her husband’s fear she would have an affair, yet ridiculed for not contributing financially to the household, Amelia suffered from poor mental and physical health as a result of her partner’s psychological and financial abuse. She added, “I think he also despises me because I am on drugs (*ART*)”. Amelia avoided taking her infant’s clinic records and her own medication home out of fear of further violence:

People were asking me where I was married because they noticed that I was not doing fine…My mother didn’t want me to go back (*to her partner’s home*). I told her that I left the child’s clinic book behind together with my drugs (*ART*). I was wondering whether I could go to the hospital and tell them that I could not access drugs because of hostility from my husband…I just came [home] so that I can observe him for some time and explore how I can escape with my drugs so that I can find peace.

-Amelia, 21 years, randomized in trial

Eucabeth, a 37-year-old woman, experienced physical, sexual, psychological and financial IPV. Her partner married another woman without her knowledge, and this caused her deep emotional pain when she found out during her pregnancy. This infidelity combined with unintended pregnancy and her HIV diagnosis during the pregnancy led to depression. Not long after delivery, her partner brought another woman with children into the mix and Eucabeth explained, “This incident hit me hard with stress. I have just woken up from my ill bed. I have been so sick and bed ridden...What is ailing me is just my heart. It is paining me a lot”. She continued to describe how her partner’s psychological abuse, including infidelity, affected her mental health:

There is no peace. When you are grounded and unable to do anything because of stress, plus taking your drugs, you feel like dying.

-Eucabeth, 37 years, randomized in trial

After complications with her physical health due to the stress she was experiencing from IPV, Eucabeth described how her partner’s psychological abuse had affected her mental health, which was affecting her HIV-related health, as she felt he was hindering her HIV treatment:

I thought, “You’ve infected me with HIV, I’ve gone through challenges, I say no!” It’s enough. I don’t have peace even as we talk….I feel no, no! I don’t have peace completely…It reached a point where I told my spouse “I want a separation; I would like to allow you freedom with your women to continue your life…I want to leave alone so that even when I continue drugs (*ART*), I ensure to reduce the HIV viral load.” At least reduce the viral load so that I can heal.

-Eucabeth, 37 years, randomized in trial

The experience of psychological IPV in the form of insults, belittling, or infidelity led to poor mental health, which undermined ART adherence for many participants.

### Financial IPV in forms of partner withholding food or money alters HIV treatment

Most women described experiencing some type of financial IPV. For many, this included partners having sole control over household finances and withholding food. Many women described living in difficult economic conditions, where they struggled to feed their children and buy basic household supplies. Sometimes their partners would provide for the household, but sometimes they would choose not to. A few women described how their partner’s control over finances directly led to them struggling with adhering to HIV treatment.

Amelia described how her partner had control over food for the household and brought home food late at night which interfered with her taking ART since it is best taken with food:

I was not getting food on time and yet I am on medication. I usually take it at 9pm. I take drugs with no food on many occasions. I have a lot of worries.

-Amelia, 21 years, randomized in trial

Furthermore, Amelia explained that as a woman who was breastfeeding, the lack of food was even more troublesome. The financial abuse she experienced, including withholding of food, contributed to her poor mental and physical health.

If you put all your hope in him (*her partner)* to bring food and just wait, it can even get to 9pm before he returns. After you retire to sleep, he then comes back at 10pm. He comes and sleeps, wakes up again tomorrow, and goes away without a word. Remember that you are breastfeeding the baby, and you are also on drugs (*ART*) and hungry at the same time. I was worried a lot and even lost weight; in fact, all my clothes do not fit me anymore. I even feel my undergarments falling off because I have a lot of worries. I am not doing well at all.

-Amelia, 21 years, randomized in trial

Sarah, a 23-year-old woman, was orphaned as a young child, became pregnant as a very young adolescent, and married young for financial security. She experienced physical, sexual, psychological, and financial IPV as well as controlling behaviors from her husband. Completely socially isolated, Sarah described how her partner did not financially support her while she was sick, did not provide money for her to buy medication, and she required permission from him to leave the house (physical IPV was the consequence of not getting permission). She described how her partner did not support her financially for her to take care of her health:

P (Participant): I struggle on my own to get drugs or I go to the hospital alone.

I (Interviewer): He doesn’t care that his wife is sick?

P: It doesn’t bother him. He is just there. Even when you ask for Kshs.10 (*a small amount of money*) for buying drugs, he says “Where have you seen it?” You have to struggle like a woman or even ask people [for] Kshs.10 to buy drugs. If I don’t get [it], I go to [the] government hospital.

-Sarah, 23 years, not randomized in trial

Furthermore, Sarah’s partner did not provide enough money for transportation to the clinic where she collected her ART. As Sarah described, “He would not [give] enough [money] to take me all the way to the clinic.” Her partner’s withholding of money created additional challenges for her to access HIV care and treatment.

In the context of food insecurity and poverty, financial IPV made it more challenging for these women to take care of their health, including HIV. For pregnant and breastfeeding women, this was doubly challenging due to the physical demands of these stages.

### IPV in the form of sabotaged treatment or partner control over access to HIV care

Women described how their partners directly sabotaged their ART or insisted they discontinue ART, and how their partner’s control led to difficulty with ART adherence and HIV care.

#### Partner sabotage of ART.

Many women described how their partners did not accept their HIV status and even insulted them for it. A few women directly described how their partner’s lack of support for ART, or even direct sabotage of ART, made ART adherence more challenging. In addition to the HIV-related insulting and ridiculing she experienced from her partner, Kioni described how her partner disposed of her ART and did not support her ART use in general after the initial diagnosis period. This lack of partner support and direct sabotage of medication contributed to her poor ART adherence, including missed ART days.

P: Sometimes I can fail to adhere because when I am taking drugs (*ART*), he ridicules me. When I go to pick the drugs and he sees them, he wants to take those drugs and throw them away. I have told him that if he does not want to know his status then he should allow me to continue with my medication…

I: Has he ever disposed of your drugs?

P: Yes, he has done so twice.

-Kioni, 23 years, not randomized in trial

Eucabeth explained how her partner made her ART adherence more difficult during pregnancy. As she described, he did not support her taking ART and told her to stop taking it:

I: Some women say it is hard to take medication every day and at the right time each day. During pregnancy, how difficult was it for you to take your HIV medications every day?

P: Very difficult! It was very heavy for me. My spouse even asked me to stop taking the drugs, but I sat down and reflected, “He is telling me to stop yet he is the one who has brought these problems. He says don’t take [ART], yet he is okay, and he is the one who is continuing with marrying more women. It is better I take care of my life.” …He wants me to die early so that I leave my children...I realized if I follow that I will be the one to suffer.

-Eucabeth, 37 years, randomized in trial

Lack of partner support for ART, including telling women to stop ART and directly throwing ART away, was a hindrance to optimal ART adherence for these women. These actions were an extension of their partner’s refusal to fully accept women’s HIV diagnoses.

#### Partner controlling movements to clinic.

Many women described experiencing controlling behaviors from their partners, but some women described how partner control directly affected their attending clinic visits for HIV care and treatment. Sarah described how if her partner did not grant permission for her to leave, including for HIV-related care, she would have to remain at home:

It is either I am going to pick drugs (*ART*) or I am going to my own business. It is those two issues he is suspicious about. So, I decide to tell him the truth so that if he gives me permission, I go and if he does not, I remain…If I just go without permission, I will be beaten.

-Sarah, 23 years, not randomized in trial

A 40-year-old woman, Duni, described how her partner had beaten her when he found out she was going to the clinic, but she proceeded to go despite his response, and he followed her there. When she was diagnosed with HIV at the clinic, she told her husband about the diagnosis, but due to her fears of his reaction and his controlling behaviors, chose not to disclose that she had started ART. She was afraid of conflict if he found out she had started ART without his permission.

After leaving the hospital, I came with the drugs (*ART*) but I did not tell him (*her partner*). I [told] him that I was tested and found to be infected. He became very angry and wanted to know which infection I was talking about. I told him that I was infected with HIV…I informed him that they had offered to give me medication, but I told them (*the nurses*) that I needed to come with him (*her partner*) so that I can be given the drugs with him.

-Duni, 40 years, randomized in trial

In these stories, we saw how controlling behaviors and physical IPV were interrelated. The controlling behaviors experienced by these women from their partners made ART adherence and attending HIV care significantly more challenging.

### Safety behaviors to avoid IPV have unanticipated consequences for HIV care and treatment

To avoid IPV or IPV escalation, some women enacted strategies such as HIV status non-disclosure and leaving the home after severe physical IPV. However, as a few women described, this led to challenges with ART adherence.

#### Non-disclosure to partners.

Most women described how they had disclosed their HIV status to their partner. However, two women described how they did not disclose their HIV status to their partner and how this directly affected their HIV treatment, or ART for their baby, due to having to hide their status.

In addition to the IPV Sarah experienced, her HIV care was complicated by the fact that she had not disclosed her HIV status to her partner. Sarah had to find times when her partner was not home to take her ART. She described how the non-disclosure made it more difficult for her to give her baby ART since her partner did not know about the HIV:

I: It is a challenge [to give your baby ART] because he (*partner*) asks you what that is?

P: Yes…

I: What about when you want to take your drugs (*ART*)?

P: I take my drugs in the evenings. I normally go to his mother’s place and take my drugs when I remain in the house alone. That is for the drugs I take at night. I also do in the mornings when he has gone to the farm because I do not go to the farm.

-Sarah, 23 years, not randomized in trial

Cora, a 30-year-old woman who experienced physical, psychological, and financial IPV as well as controlling behaviors from her partner, had not disclosed her HIV status to her partner due to fear of his response. Cora was afraid he might be violent towards her and/or might leave her. She described how this added to the difficulty of taking ART, particularly postpartum, because she had to hide it from him:

At the moment, I am finding it very hard because I could be found taking drugs [and] he doesn’t know that I am on ART. He can ask, “Why are you taking drugs and you are not sick?” At times it can be hard. The time I was expectant, I would simply say these are pregnancy drugs I was given in hospital…but now, I feel it can be a bit hard…My worries are if he knows about these drugs (*ART*) and he has never known, I believe there can be violence.

-Cora, 30 years, randomized in trial

Due to fear of their partner’s response, these women strategically hid their HIV status from their partners to prevent violence escalation. However, this non-disclosure resulted in challenges with ART adherence.

#### Leaving home after physical IPV.

While many women described experiencing physical IPV, a few women directly described periods of non-adherence to ART after having to leave the marital home due to physical IPV. Nyambura, a 31-year-old woman who experienced physical, psychological, and financial IPV as well as reproductive coercion, described that, as a result of physical and psychological IPV during pregnancy, she became so depressed that she attempted suicide multiple times. Nyambura’s partner physically abused her while pregnant and after delivery. In addition, her partner heavily insulted her for having HIV and disclosed her status to others without her consent. As she described, “He repeatedly insulted me [for taking ART] until I felt I had reached a dead end. I would rather die.” Nyambura described an incident that occurred two weeks after delivery during which she was severely physically assaulted by her partner and forced to leave the home:

He removed me from the bed…[and] dragged me to the field outside…He seriously beat me…until my sister came…and said, “You want to kill my sister? You want to finish her?” He beat me and took a machete to cut me…When he stopped beating me, I left home and went to sleep at [the home of] another elderly woman who is a friend…[Then] I came back to my parents’ home…When I came home, my brother brought me to hospital and the doctor gave me a P3 form (*police form for officially reporting violence*) which he completed for assault. The case was later just dismissed like that…So because the case was dismissed, I left home and went to live with an aunt of mine. I stayed there for some time and then my spouse began calling again that I go back home. I was not living a comfortable life at my aunt’s place, my drugs (*ART*) depleted, I was so sick and almost died. The drugs I came with from home were finished, so I went to a nearby hospital where I was given a few doses and was asked to bring a transfer letter from the other facility before replenishing.

-Nyambura, 31 years, not randomized in trial

After this period, Nyambura recognized that her partner’s physical IPV which caused her to temporarily migrate from home, had a significant impact on her HIV care and health. Because of this, she explained that that she was steadfast on adhering to ART when she was able.

Isla, a 35-year-old woman, experienced physical, psychological, and financial IPV as well as reproductive coercion from her partner. She described how she and her children went hungry when her partner did not provide food which stressed her during pregnancy. In addition, she endured infidelity from her partner and was severely physically abused. Isla described an incident that occurred during which she suffered physical IPV and then left the home for a couple weeks without her HIV medication:

When I was pregnant with my first child, he assaulted me, and I ran away…I only managed to take my clinic card. I stayed out for two weeks before taking medication (*ART*)…I was at my aunt’s place, but I did not come along with the medication…When I was giving birth, my in-law remarked that it (*the physical violence*) was the reason why I had a stillbirth. Probably he beat me and injured the child and that is why the problems arose. He assaulted me very badly and I ran away because I was almost giving birth. I knew that with the card I could go to a different hospital when I needed to give birth. I ran away and left my drugs behind.

-Isla, 35 years, not randomized in trial

Isla described how she prioritized her baby’s immediate health in that instance over her HIV-related health as she was very close to the expected delivery date. In these cases of severe physical IPV, women were forced to leave their homes to protect themselves. Sometimes they were unable to grab necessities, such as a clinic card or HIV medication, before leaving the home.

### Resilience pathway of commitment to HIV treatment for children and themselves

Despite living with multiple types of IPV, suffering from poor mental health, living with food insecurity, and/or being economically disempowered, nearly all women described their commitment and dedication to taking ART. Women recognized that they needed to maintain good ART adherence to sustain good health, even though they experienced challenges, including IPV, which made it more difficult. Asilia, a 25-year-old woman who experienced physical, psychological, and financial IPV, as well as controlling behaviors and reproductive coercion, described how she was dedicated to taking ART despite IPV:

I: Some women find it a challenge to take HIV medication because of the differences they have with their partners. Have you experienced something similar?

P: I cannot fail to take medication because I am the one who will benefit from it. Even if I stop taking medication, the other person continues with his life. Therefore, I take medication without fail.

-Asilia, 25 years, randomized in trial

Eucabeth described the desire to maintain good ART adherence to keep herself healthy to take care of her children:

I: Did you find it easier or harder, or no difference, to take your HIV medications every day after the baby was born?

P: I am used [to the daily regimen]. Before it was a heavy burden, but now I am used [to it], especially after deciding that the way my life is important to my parents, so is my child’s life important to me. So, why should I lose my life and make my children suffer?

-Eucabeth, 37 years, randomized in trial

Some women described how they found ways to cope with IPV and strategies to maintain their HIV-related health. As described, Eucabeth told her partner she wanted a separation so that she could heal her mental health and improve her HIV-related health. To avoid IPV and stress, Eucabeth described how she sidestepped asking her partner for money to travel to the clinic and figured out other ways to get there. Several women described how they would strategically cope with financial IPV by saving money on their own to buy household items, food, or pay for transport to the clinic. Achieng, a 23-year-old woman, described how she would save money on her own for upcoming clinic appointments because she experienced financial IPV and could not rely on her husband financially. In the cases of partner non-disclosure as described, Sarah and Cora found creative and safe ways to take their ART and maintain their HIV-related health.

Awino, a 36-year-old woman, who experienced physical, sexual, psychological, and financial IPV, described how she avoided worrying and coped with the stress she experienced as a result of IPV in order to avoid poor mental health and poor ART adherence:

If I allowed myself to worry, I think my life would have been cut short because I would have defaulted on taking drugs and my health would have therefore deteriorated. Eventually I would have died.

-Awino, 36 years, randomized in trial

In this study, we saw examples of the strength and resiliency of women in their commitment to their children, to their own HIV-related health, and to their ART adherence. Despite IPV and other challenges, women were dedicated to maintaining good ART adherence.

## Discussion

Postpartum women living with HIV and IPV described experiencing multiple types of partner violence in their relationships, including physical, sexual, psychological, and financial. Reproductive coercion, male controlling behaviors, and partners withholding or sabotaging HIV treatment also emerged as salient forms of IPV in this sample. Many women described a link between IPV and their challenges with perinatal HIV care and treatment, with direct and indirect pathways tying these conditions together. There was also a resilience pathway illustrated by women whose persistence with HIV care and treatment overcame their exposure to IPV.

This study identified ways in which psychological IPV led to difficulty with ART adherence among WLH due to negative effects on mental health, including depression and anxiety. Additionally, we found financial IPV and withholding food led to challenges with taking ART and maintaining good HIV care. Our result on mental health is supported by existing qualitative and quantitative evidence which suggests the mediating role of poor mental health in the association between IPV and poor ART adherence among pregnant and postpartum women [10, 30–32]. Studies from South Africa and Zambia found IPV worsened HIV-related health via poor mental health such as depression [10, 30–32], anxiety [10, 30–32], and post-traumatic stress disorder [10]. In qualitative studies from Zambia, pregnant WLH described how male partners were ‘weaponizing’ women’s HIV-positive status through psychological and financial abuse [30, 33]. This included degrading women’s self-worth through accusations of infidelity, blaming and belittling women for having HIV, and ignoring women. This psychological abuse and lack of support negatively affected their mental health and made ART adherence more difficult, including for their babies. This Zambian qualitative research also found when male partners did not approve of or accept women’s HIV status, they would withhold money and food [30, 33]. One of these Zambian studies found how the lack of support of her HIV status from one pregnant woman’s partner led to financial IPV which then led to poor mental health [30]. Thus, it is plausible that the pathways between psychological IPV, financial IPV, mental health, and poor HIV care and treatment can be entangled and fluid. Additionally, a qualitative study among WLH from Tanzania found male partners would not provide women with money to travel to the HIV clinic or buy food and this impaired their HIV care and treatment [44]. Financial IPV can affect women’s HIV care and treatment through multiple avenues, including withholding food, withholding money to access HIV services or travel to clinics, and/or through negative effects on mental health.

Results from our study showed WLH experienced IPV in a unique form. Partners directly sabotaged women’s ART or insisted they discontinue ART, and partner control led to difficulty with ART adherence and HIV care. This aligns with previous qualitative research from Zambia which found how one pregnant WLH reported that her partner wanted her to stop taking ART and blamed her for acquiring HIV, including accusing her of infidelity [30]. She stopped taking ART because her partner did not like it and she feared him. A qualitative study from Tanzania found how some male partners got angry when women took their ART and disposed of women’s medication [44]. This led to women running out of medication and poor ART adherence. A South African qualitative study, as well as the qualitative study from Tanzania, align with our results as they found that partner control limited women’s ability to attend HIV care since partner’s controlled their movement and did not want women attending clinic [31, 44].

Our study found that navigating violent relationships can have unintended consequences on HIV care and treatment, through non-disclosure to partners to prevent violence escalation or through leaving the home after severe physical IPV. HIV status nondisclosure can lead to challenges in women’s HIV care and treatment due to fear of their partner’s reaction, including violence, and make it harder for women to adhere to ART due to having to hide it, lie about what it is for, or seek HIV care without their partner’s knowledge [30, 31, 45]. A scoping review of violence against women and engagement in HIV care and treatment found qualitative evidence that violence against women could lead to poor ART adherence through partner non-disclosure [45]. Our findings align with previous qualitative research from Zambia and South Africa which found pregnant WLH were afraid to disclose their HIV status to their partner due to violence, and non-disclosure led to challenges with HIV care and treatment [30, 31]. Similar to our study, their narratives were closely tied to gender inequality, male controlling behavior, and HIV stigma. Non-disclosure can be a strategic choice for many reasons, primarily related to fear of partner response or partner stigma, and this fear may be heightened in relationships with past violence. We could not find similar evidence in the literature regarding our finding of challenges with HIV treatment after leaving the home subsequent to physical IPV. However, a previous qualitative study from the same geographical area found some pregnant women left their home to move back to their maternal home in response to physical violence to prevent further violence [46]. Sometimes these women would have to return home due to lack of economic resources of maternal families. However, that study included WLH as well as women without HIV and did not assess the impact on HIV care and treatment. Thus, our finding is a novel addition to the evidence base on pathways between IPV and HIV care and treatment in perinatal WLH.

Despite living with multiple types of IPV, many women in this study described their dedication to taking ART. This finding is supported by qualitative research from Zambia, South Africa, and Tanzania which also found women were resilient and committed to ART despite living with IPV [30, 31, 44]. Women who had not disclosed their HIV status to their partners found creative ways to seek HIV care and safely take their ART [30, 31, 44], and the desire to protect one’s health for the sake of their children was found as a protective pathway [31, 44].

Addressing IPV in maternal health settings such as antenatal and postnatal care should be a policy priority for HIV policy and programming. The results of this study suggest women living with HIV and IPV experience multiple forms of IPV and multiple barriers to maintaining optimal HIV care and treatment. For pregnant and breastfeeding WLH, addressing IPV is important for efforts to end vertical transmission of HIV as well as to improve maternal and infant health. Additionally, offering access to mental health and financial assistance services may improve women’s health and HIV outcomes. During this time in a woman’s life there may be increased opportunity for intervention. The repeated interactions with the healthcare system through antenatal and postnatal care represent a ‘window of opportunity’ to address IPV and vertical transmission of HIV.

Future quantitative research is needed to assess the impact of different forms of IPV on HIV care and treatment outcomes, such as ART adherence and viral suppression, as this represents a gap in the literature. To our knowledge, there is not any existing research evaluating interventions for improving HIV treatment outcomes for pregnant and/or postpartum women living with HIV in Kenya, or any other sub-Saharan African country, after experiencing physical IPV and leaving the home. The pilot Jamii Bora study, a home-based intervention including health education, relationship-building skills, and couples HIV Testing and Counselling in Kenya, found pregnant women living with HIV in the intervention group had three times the odds of perfect ART adherence at three months postpartum compared to the standard of care group [47]. However, the pilot study did not include women experiencing severe IPV and did not report on any IPV-related measures. Results from the parent Jamii Bora study are forthcoming, but briefly, the home visiting intervention was shown to reduce viral load in couples compared to couples in the standard of care group [48]. Future research is needed to find ways to better assist women with HIV-related health after experiencing physical IPV and leaving the home.

### Strengths and limitations

There are some limitations to consider. Women were originally recruited from ANC clinics, so this study did not include women who do not seek healthcare. For women living with HIV, the Jamii Bora study only included women who were currently living with their partner and were in an HIV discordant relationship or unknown HIV status of the male partner, thus we were unable to capture the experience of women who do not live with their partners or are in HIV+ concordant relationships. Approximately one-third of participants in this sub-study were in one of the Jamii Bora intervention arms, and two-thirds were in the non-randomized group, and this could have affected the results. There may have been unequal dynamics between the interviewer and participants in terms of class and/or education, though we aimed to reduce this limitation through constructivist interview approaches and ongoing interviewer supervision. The richness of data collected suggests the comfort women felt sharing their stories with the researcher. Additionally, the researcher was fluent in the local languages and grew up near study communities. A positionality limitation is the write-up of findings was led by the first author, who is a white, English-speaking, US researcher. This was overcome through ongoing input from the second author, a black Dholuo-speaking, Kenyan researcher. Furthermore, team-based input was incorporated at key moments of interview guide creation, thematic codebook development, data collection, interpretation, and presentation of findings. Because this qualitative study used purposive sampling from an existing trial in a rural area of Kenya it may not be generalizable for other populations. Nevertheless, the dynamics highlighted here are worth elucidating in future studies, particularly since many of the findings echo research from other African settings.

## Conclusions

Pregnant and postpartum women living with HIV and IPV in southwestern Kenya experience multiple types of IPV. Women described IPV as detrimental to maintaining optimal HIV care and treatment through multiple pathways. To end vertical transmission of HIV and improve maternal and infant health, efforts to address IPV within maternal health settings should be prioritized in HIV policy and programming.

## Supporting information

S1 File Inclusivity in global research. (DOCX)
